# KIF5B-RET fusion kinase promotes cell growth by multilevel activation of STAT3 in lung cancer

**DOI:** 10.1186/1476-4598-13-176

**Published:** 2014-07-21

**Authors:** YingYing Qian, Shoujie Chai, Zuyu Liang, Yongfang Wang, You Zhou, Xia Xu, Chenchen Zhang, Min Zhang, Jingxing Si, Feiteng Huang, Zhangdan Huang, Wei Hong, Kai Wang

**Affiliations:** 1 Department of Respiratory Medicine, Second Affiliated Hospital, School of Medicine, Zhejiang University, Hangzhou 310009, China; 2 Department of Medical Oncology, Zhejiang Cancer Hospital, Hangzhou 310022, China

**Keywords:** KIF5B-RET, Lung cancer, Cell growth, STAT3 pathway

## Abstract

**Background:**

Lung cancer in nonsmokers tends to be driven by a single somatic mutation or a gene fusion. KIF5B-RET fusion is an oncogene identified in non-small cell lung cancers. In this study, we verified the oncogenic activity of KIF5B-RET fusion and investigated how KIF5B-RET activates the specific signaling pathways for cellular transformation. We aimed to provide a basis for the further development of the therapy for KIF5B-RET positive lung cancer patients.

**Methods:**

RT-PCR was used to screen for KIF5B-RET fusions in Chinese lung cancer patients. To verify the oncogenic activity of KIF5B-RET kinase in lung cancer cells, we manipulated its expression genetically followed by colony formation and tumor formation assays. The mechanism by which KIF5B-RET kinase induces proliferation was investigated by western blot, coimmunoprecipitation, and administration of RET, MAPK and STAT3 inhibitors.

**Results:**

Our study identified a KIF5B-RET fusion in Chinese NSCLC patients and demonstrated that KIF5B-RET transfected cells showed a significantly increased proliferation rate and colony-forming ability. Furthermore, we found that KIF5B-RET fusion kinase induced multilevel activation of STAT3 at both Tyr^705^ and Ser^727^, and KIF5B-RET-STAT3 signaling related inhibitors repressed the proliferation and tumorigenicity of lung cancer cells significantly.

**Conclusions:**

Our data suggest that KIF5B-RET promotes the cell growth and tumorigenicity of non-small cell lung cancers through multilevel activation of STAT3 signaling, providing possible strategies for the treatment of KIF5B-RET positive lung cancers.

## Background

Lung cancer is a common malignancy and is the leading cause of cancer deaths in the world, and non-small cell lung cancer (NSCLC) is the most common tumor type [1]. NSCLC in nonsmokers tends to be driven by a single somatic mutation or a gene fusion [2], such as mutated epidermal growth factor receptor (EGFR) [3], v-Ki-ras2 Kirsten rat sarcoma viral oncogene homolog (KRAS) [4], the echinoderm microtubule associated protein like 4 (EML4) and anaplastic lymphoma receptor tyrosine kinase (ALK) genes (EML4-ALK) [5], etc. These have been proven to be “driver” genes in some subgroups of lung cancers. Recently, NSCLCs were reported to harbor novel gene fusions involving ROS1 [6] and RET [7,8]. RET is the receptor for members of the glial cell line-derived neurotrophic factor family (GDNF) [9,10]. The RET gene is located on chromosome 10 and encodes a receptor tyrosine kinase, and the oncogenic potential of this gene product has been suggested in several tumors, especially in thyroid cancers [11].

KIF5B-RET is a novel fusion gene of the kinesin family member 5B gene (KIF5B) and the rearranged during transfection gene (RET) resulting from the chromosome inversion inv (10) (p11; q11). It was first identified in non-smoking Korean man as adenocarcinoma by whole-genome and transcriptome sequencing [7]. KIF5B-RET is reported in a low percentage of lung cancers and is more frequent in non-smokers, in patients with adenocarcinoma, and exists exclusively with other mutations, such as EGFR, Kras, Braf, ErbB2 or EML4/ALK fusions [8,12,13]. It has been demonstrated that KIF5B-RET fusions may be oncogenic drivers and potential targets for existing small-molecule tyrosine kinase inhibitors (TKIs). However, it is unclear how KIF5B-RET activates the specific signaling pathways for cellular transformation. The incomplete understanding of its carcinogenic mechanisms leads to difficulties in selecting targeted treatment.

Signal transducer and activator of transcription-3 (STAT3) is a member of a gene transcription protein family that mediates a variety of biological processes including cell proliferation and carcinogenesis [14]. Constitutive STAT3 activation has been found in multiple types of tumors [15-18]. Conventional STAT3 activation mainly consists of phosphorylation on a single tyrosine residue Tyr^705^ resulting in dimerization of the STAT1/3 transcription factors, translocation into the nucleus and transcriptional activation of target genes [19].

Previous studies have demonstrated that STAT3 is required for efficient cellular transformation by an array of well-characterized oncogenes including EGFR [20], RAS [21], ALK [22], and even RET related oncogenes such as RET-MEN2A/2B [23], RET-FMTC [24] and RET/PTC [25,26]. Phosphorylation on the Ser^727^ residue enhances the transcriptional activity of STAT3. Different cellular systems may affect STAT3 Tyr^705^ or Ser^727^ phosphorylation as different protein kinases may be implicated. It is reported that RET-MEN2A mutant RETC634R induces both Tyr^705^ and Ser^727^ phosphorylation of STAT3 for cellular transformation in a process independent of JAKs and Src [23]. By contrast, the FMTC-associated mutants RETY791F and RETS891A implicate Src and JAKs in constitutive activation of STAT3 only in Tyr^705^[27]. While thyroid-specific rearrangement of RET/PTC phosphorylating STAT3 Tyr^705^ requires the intrinsic kinase activity of RET/PTC and JAKs, and c-Src kinase are not involved in the RET/PTC-mediated activation of STAT3 [28].

Here, we screened for known KIF5B-RET fusions in Chinese NSCLC patients using real-time polymerase chain reaction (RT-PCR) and corroborated the oncogenic activity of this fusion kinase by colony formation and tumor formation assays. And we explored the hypothesis that STAT3 may be involved in the signaling triggered by KIF5B-RET. We aimed to provide a basis for the further development of the therapy for KIF5B-RET positive lung cancer patients.

## Results

### A KIF5B-RET fusion gene is screened in Chinese LAD patients

**Figure 1:**
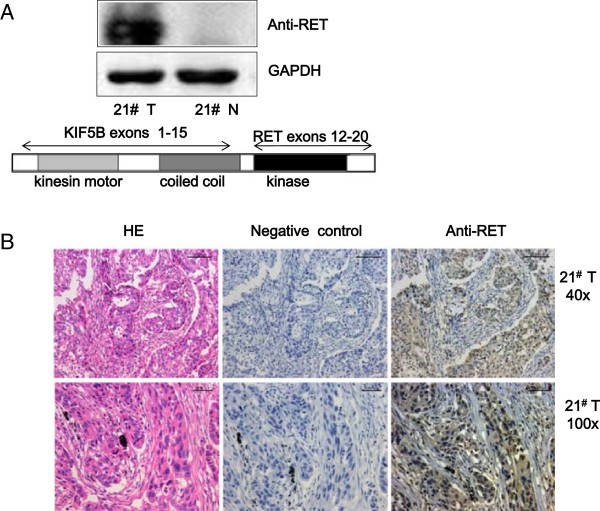
**KIF5B-RET fusion gene is identified in a Chinese LAD patient. A**. Expression of the KIF5B-RET fusion protein is shown in the positive tumor sample. Western blot analysis showed an approximately 108 kDa but not 170 kDa (the RET protein) band. **B**. Immunohistochemical analysis of KIF5B-RET expression in the positive tumor tissue. The tumor cells were arranged in solid nests and revealed moderate cytoplasmic expression of KIF5B-RET fusion protein.

We performed RT-PCR assay for the KIF5B-RET fusion genes in 100 Chinese LAD (Lung adenocarcinoma) patients. One of samples had PCR products, suspected as KIF5B-RET fusion genes, while matched adjacent normal lung tissue had no band, suggesting that the translocation was somatic (Additional file 1: Figure S1.A). KIF5B-RET transcripts were detected in 1 of 100 tumors (about 1.0%). None of the 7 lung cancer cell lines that we examined harbored the fusion transcript (data not shown). From the direct sequencing of the PCR products, it showed junctions between exon 15 of the KIF5B gene and exon 12 of the RET gene (Additional file 1: Figure S1.B). We subsequently measured the protein expression of KIF5B-RET in the positive tumor tissue. Based on the mRNA sequence of KIF5B-RET, the predicted protein is approximately 108 kDa. Western blot analysis using an anti-RET antibody revealed a protein band, of which the size corresponded to the predicted value, inferring the expression of KIF5B-RET fusion protein (Figure 1A). Imunohistochemical analysis performed on the paraffin sections of the positive tumor tissue revealed moderate, cytoplasmic, non membranal, KIF5B-RET expression in tumor cells (Figure 1B). Since RET protein had low expression in this sample, the staining implicated expression of the fusion proteins.

This positive case was a poorly differentiated nodule subtype LAD. The patient was a countrywoman aged 58 years, and had no smoking history. The mass measured 4 cm in greatest dimension, and the right lung pleura and the bronchus were involved, but no lymph node metastasis or distant metastasis (pathologic stage I b). RET expression was observed as moderately intense cytoplasmic staining but no obvious perinuclear or paranuclear accentuation. The tumor cells also had strong expression of TTF1 and Napsin A. Characteristics are listed in Additional file 1: Table S1 and Additional file 1: Table S2. The clinicopathological characteristics of this case were consistent with previous researches [29], namely, more poorly differentiated tumors, younger (<65 years), non-smokers, and solid subtype.

### KIF5B-RET fusion kinase is constitutively activated and enhances cell proliferation

**Figure 2:**
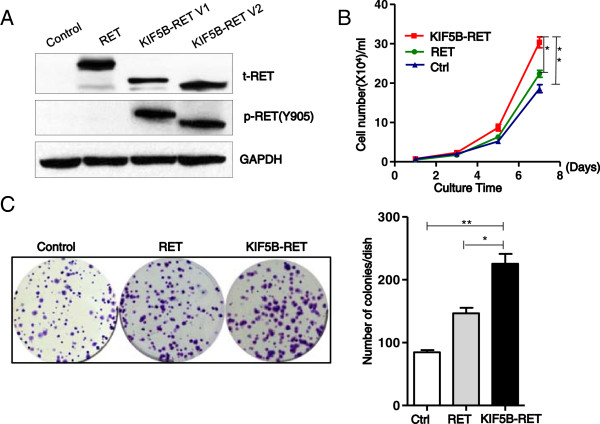
**KIF5B-RET fusion kinase is constitutively active and enhances cell proliferation *in vitro. *A**. KIF5B-RET fusion kinase is constitutively active. A549 lung cancer cells were transfected with an empty vector, wild-type RET (RET) or KIF5B-RET variants1 or 2 expression plasmids, and cultured without GDNF for 48 hour. The level of phosphorylated Tyr905 (pTyr905) RET in cell lysates was analyzed by western blots. **B**. Expression of KIF5B-RET fusion protein enhances positive cell proliferation *in vitro*. A549 cells carrying KIF5B-RET,RET or empty vector were seeded in 6-well plates and cultured for 7 days, and cell numbers were obtained at the indicated time (^*^P < 0.05, ^**^P < 0.01, Student’s t test). **C**. Expression of KIF5B-RET fusion protein enhances the colony forming ability of positive cells *in vitro*. A549 cells carrying KIF5B-RET, RET or empty vector were seeded in 6-well plates, and cultured for 14 days. The total number of colonies, each containing more than 40 cells, were determined (^*^P < 0.05, ^**^P < 0.01, Student’s t test).

**Figure 3:**
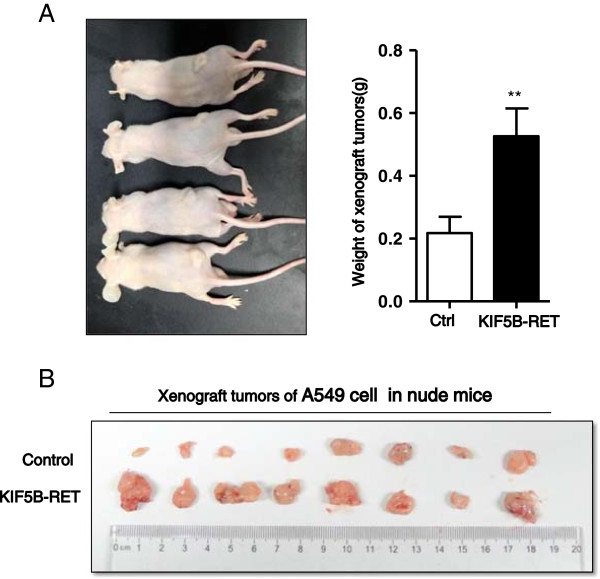
**Expression of KIF5B-RET fusion protein promotes the growth of positive cell xenograft tumors *in vivo*. A**. 1 × 10^6^ control and enhanced KIF5B-RET expressing A549 cells were separately subcutaneously injected in the left and right flanks of mice, and the mice were shown 1 month later. **B**. The weight of xenograft tumors was recorded and analyzed (n = 8, ^**^P < 0.01, Student’s t test).

It was predicted that the N-terminal portion of the KIF5B coiled-coil region, retained in all variants, has the ability to dimerize through two coiled-coil structures, similar to the KIF5B-ALK fusions [30]. Consistently, when the KIF5B-RET variant 1 and 2 were exogenously expressed in A549 human lung cancer cells, RET Tyr905 was phosphorylated in the absence of serum or GDNF stimulation, indicating an aberrant activation of RET kinase by fusing with KIF5B (Figure 2A). To determine whether KIF5B-RET directly affects the proliferative and colony-forming abilities of NSCLC cells, human lung cancer A549 cells were infected with KIF5B-RET-expressing, wild type RET expressing, or control lentivirus. After blasticidin selection, the cell proliferation was measured with cell counting and the number of colonies was scored. The KIF5B-RET transfected cells showed a significantly increasing proliferation rate when compared with the RET transfected or control cells (Figure 2B). Similarly, compared with control and RET-expressing cells, the enforced expression of KIF5B-RET in A549 cells also caused an increase in colony number and size (Figure 2C). Consistent with *in vitro* observations, we also confirmed that the enforced expression of KIF5B-RET caused a significant increase in A549 xenograft tumor weight in nude mice compared with control (KIF5B-RET group *vs* control group: 0.53 ± 0.2 g *vs* 0.22 ± 0.15 g, ^***^P < 0.001; Figure 3). All of these findings corroborate that the KIF5B-RET fusion kinase promotes the growth of lung cancer cells both *in vitro* and *in vivo*.

### Signaling pathways involved in the proliferation of KIF5B-RET positive cells

**Figure 4:**
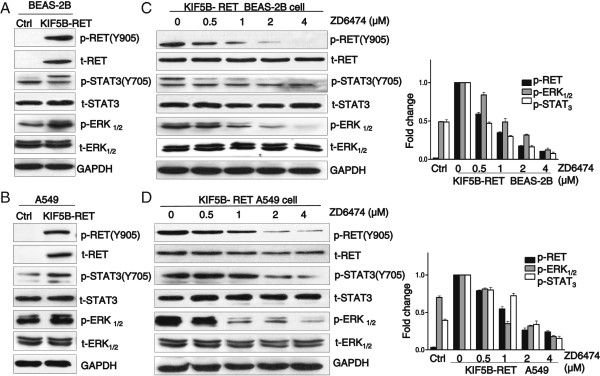
**Signaling pathway of the proliferation is involved in KIF5B-RET positive cells. A & B**. KIF5B-RET positively regulates the activation of the total STATs and ERK signaling pathways in positive cells. Control and enhanced KIF5B-RET expressing BEAS-2B **(A)** or A549 **(B)** cells were lysed and analyzed for phosphorylated Tyr^705^ STAT3 and Thr^202^/Tyr^204^ ERK_1/2_ by western blots. **C & D**. The suppression of KIF5B-RET kinase activity by ZD6474 affects both STATs and ERK signaling pathways. Positive BEAS-2B or A549 cells were treated with ZD6474 at different doses for 48 h. Cells were lysed and analyzed by western blots for indicated marker antibodies. GAPDH was used as a loading control.

It has been shown that KIF5B-RET fusion kinase promotes cell proliferation of lung cancer, then the phosphorylation levels of proliferation related signaling molecules was investigated by measuring the enforced expression of KIF5B-RET in A549 and Beas-2b cells. We found that ERK and STAT3 signaling pathways were aberrantly activated in enhanced KIF5B-RET-expressing Beas-2b (Figure 4A) or A549 cells (Figure 4B). Moreover, the KIF5B-RET-induced phosphorylation of ERK and STAT3 was also reduced by ZD6474 treatment in a similar dose response to RET phosphorylation (Figures 4C& 4D). Taken together, ERK and STAT3 signaling pathways may be involved in mediating the effect of KIF5B-RET in promoting cell proliferation.

### KIF5B-RET activates STAT3 both Tyr^705^ and Ser^727^ at different levels in positive cells

**Figure 5:**
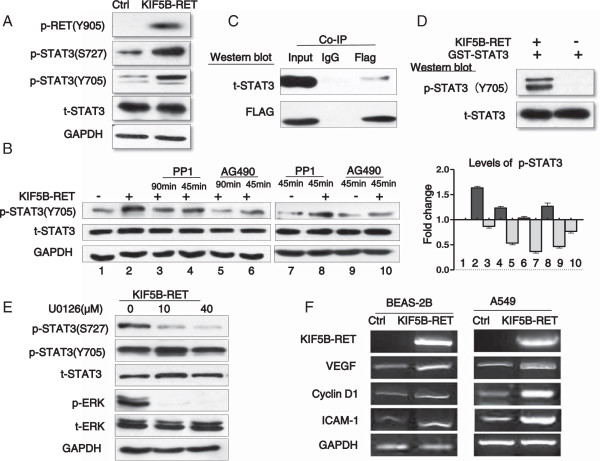
**KIF5B-RET phosphorylates and activates STAT3 at different levels in positive cell. A**. KIF5B-RET phosphorylates and activates STAT3 both Tyr^705^ and Ser^727^ in positive cells. Western blots were performed on 293 T carrying KIF5B-RET or empty vector cells lysates using the indicated antibodies. **B**. JAK2 and c-Src kinase are involved in KIF5B-RET mediated-STAT3 Tyr^705^ phosphorylation. 293 T cells carrying KIF5B-RET were pretreated with 15 μM AG490 (Jak2 inhibitor) or 5 μM PP1 (Src inhibitor) for the indicated times before cell lysis. Western blot was then performed using the indicated STAT3 antibodies. **C**. KIF5B-RET directly interacts STAT3 in positive cells. Enhanced KIF5B-RET expressing 293 T cell lysates were co-immunoprecipitated with control IgG or FLAG antibody. The immunoprecipitates were then subjected to western blot analysis with the indicated antibodies. **D**. KIF5B-RET directly phosphorylates STAT3 Tyr^705^. A bacterial-expressed GST- STAT3 fusion protein was incubated in vitro with KIF5B-RET IG that had been immunoprecipitated from 293 T carrying KIF5B-RET cell lysates. Western blots were then performed on the reaction mixtures using the indicated STAT3 antibodies. **E**. KIF5B-RET also induces a Ras/ERK_1/2_/STAT3 Ser^727^ pathway. Enhanced KIF5B-RET expressing 293 T cells were treated overnight with U0126 (MEK inhibitor), and cell lysates were analyzed by western blot using the indicated antibodies. **F**. Expression of cyclin D1, VEGF, and ICAM-1 in KIF5B-RET positive cells. Two clones of the A549 and BEAS-2B cells, which exhibited stable KIF5B-RET expression, were analyzed cyclin D1, VEGF and ICAM-1 expression by RT-PCR, the products were separated by gel electrophoresis and visualized using ethidium bromide staining.

To further investigate the role of STAT3 in mediating the function of KIF5B-RET in cell growth, we analyzed STAT3 phosphorylation in 293 T cells expressing KIF5B-RET. As shown in Figure 5A, the expression of KIF5B-RET fusion kinase increased STAT3 phosphorylation on both Tyr^705^ and Ser^727^. As the JAK and c-Src tyrosine kinases are well known as upstream kinases for the phosphorylation of the Tyr^705^ residue in STAT3, we next asked whether these kinases were still involved in KIF5B-RET-induced Tyr^705^ phosphorylation of STAT3. Indeed, both a JAK2 inhibitor (AG490) and a c-Src inhibitor (PP1) inhibited STAT3 on Tyr^705^ phosphorylation in various degrees. However, more interestingly, after treating with AG490 or PP1, the KIF5B-RET-expressing A549 cells still expressed a higher level of STAT3 Tyr^705^ phosphorylation compared to the control (Figure 5B), indicating that apart from the upstream JAK and c-Src kinases, KIF5B-RET may also directly phosphorylate and activate STAT3 Tyr^705^. We next performed a co-immunoprecipitation assay to confirm whether KIF5B-RET binds to STAT3. KIF5B-RET-FLAG expressing 293 T cell lysates were incubated with FLAG antibody, and the immunecomplexes were then purified, separated by SDS-PAGE, and analyzed with western blot. We found that STAT3 was present in the protein complex immunoprecipitated by the FLAG antibody (Figure 5C). To determine whether STAT3 is the direct substrate of KIF5B-RET, purified GST-STAT3 protein was incubated with purified activated KIF5B-RET protein from immunoprecipitation, and the result showed that activated KIF5B-RET phosphorylated STAT3 at Tyr^705^ site (Figure 5D). Taken together, these observations suggested that STAT3 is likely a direct substrate of KIF5B-RET in positive NSCLC cells, and that KIF5B-RET induced cell proliferation may be mediated, at least in part, through its phosphorylation of STAT3 Tyr^705^ directly. In addition, our previous experiments show KIF5B-RET co-activated ERK and STAT3 signaling pathways in positive cells, and the MEK_1/2_ inhibitor (U0126) attenuated the KIF5B-RET-induced ERK_1/2_ and STAT3 Ser^727^ phosphorylation, but not Tyr^705^ phosphorylation (Figure 5E), suggesting that KIF5B-RET-induced STAT3 Ser^727^ phosphorylation was partly mediated through the Ras/Raf/MEK_1/2_/ERK_1/2_ pathway. Since cyclinD,VEGF,and ICAM-1 are major targets of STAT3, and are involved in cell proliferation and invasion, we analyzed whether KIF5B-RET affected expression of these genes. As shown in Figure 5F, the expression of cyclinD1, VEGF and ICAM-1 was higher in KIF5B-RET positive cells than in parental A549 and BEAS-2B cells. Together, KIF5B-RET fusion protein induces multilevel activation of STAT3 which may target cyclinD1, VEGF and ICAM-1 and play a key role in oncogenesis.

### Down regulation of KIF5B-RET-STAT3 signaling suppresses the proliferation of positive lung cancer cells

**Figure 6:**
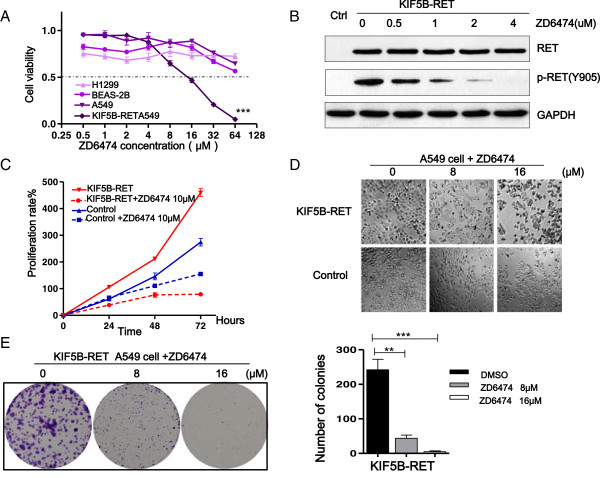
**The suppression of KIF5B-RET kinase activity reduces the proliferation of positive lung cancer cells. A**. Specific effects of ZD6474 on KIF5B-RET positive cells. A549 carrying KIF5B-RET cells and negative (A549, BEAS-2B and NCI-H1299) lung cell lines were seeded in 96-well plates, and then treated at different doses as indicated for 48 hours, and analyzed the IC50 by MTT assay. **B**. The suppression of KIF5B-RET kinase activity by ZD6474. The transformed KIF5B-RET A549 cells were treated either with DMSO or ZD6474, as indicated doses for 48 hours. The level of phosphorylated Tyr905 RET was analyzed by western blot. **C & D**. Effect of ZD6474 on the positive cells proliferation *in vitro*. A549 cells carrying KIF5B-RET or empty vector were seeded in 96-well plates and cultured for 72 hours, and proliferation rate was analyzed by MTT assay at the indicated time, and cellular morphology was obtained by light microscope at 48 hours. **E**. Effect of ZD6474 on the colony-forming ability of positive cells *in vitro*. A549 carrying KIF5B-RET cells were diluted and seeded in 6-well plates, treated with DMSO, ZD6474 8 μM or 16 μM for 14 days. The total number of colonies, each containing more than 40 cells, was determined (**P < 0.01, ***P < 0.001, Student’s t test).

**Figure 7:**
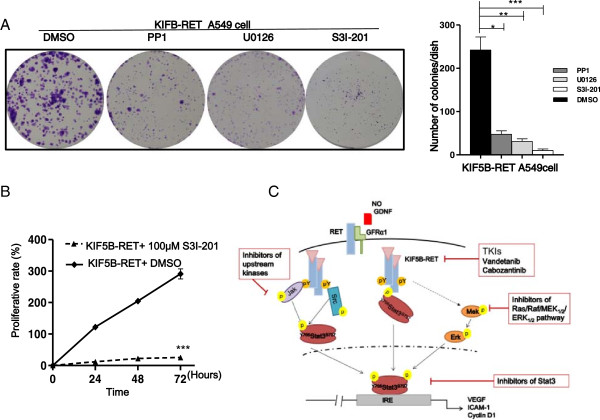
**Inhibition of STAT3 signaling reduces the proliferation of positive lung cancer cells. A**. The inhibitors U0126, PP1 and S3I-201 reduce the colony-forming ability of KIF5B-RET positive cells. A549 cells carrying KIF5B-RET were diluted and seeded in 6-well plates, treated with DMSO, U0126 (MEK inhibitor) 10 μM, PP1 (SRC inhibitors) 5 μM or S3I-201(STAT3 inhibitors) 100 μM for 14 days. The total numbers of colonies, each containing more than 40 cells, were determined. (^*^P < 0.05, ^**^P < 0.01, ^***^P < 0.001, Student’s t test). **B**. The suppression of STAT3 reduces the cell proliferation of KIF5B-RET positive cells. A549 cells carrying KIF5B-RET were seeded in 96-well plates and treated with DMSO (0.3%) or S3I-201 (100 μM) for 72 hours, and were analyzed by MTT assay at the indicated time (^***^P < 0.001, Student’s t test). **C**. Strategies to inhibit KIF5B-RET in lung cancer therapy. KIF5B-RET fusion kinase is constitutively active without GDNF stimulation. The fusion kinase induces STAT3 Tyr^705^ phosphorylation directly, and also phosphorylates Tyr^705^ indirectly through classical JAKs /STAT3 pathway, and STAT3 transcriptional activity is further enhanced by Ser^727^ phosphorylation via a MEK/ERK_1/2_ pathway. Inhibitors at different levels of KIF5B-RET-STAT3 signaling can suppress cell proliferation triggered by KIF5B-RET in lung cancer cells.

The important role of KIF5B-RET-STAT3 in promoting the proliferation of lung cancer cells led us to wonder what would happen after inhibition at any step of these signalings. To date, a few tyrosine kinase inhibitors have been shown to inhibit oncogenic RET activity, for example, ZD6474, which is a selective inhibitor of the vascular endothelial growth factor (VEGF) receprot-2 tyrosine kinase. We first assessed the IC50 values of ZD6474 in four different NSCLC cell lines (Beas-2b, NCI-H1299, A549, and KIF5B-RET-expressing A549 cells) (Figure 6A). The IC50 value was 14.20 μM in KIF5B-RET-expressing A549 cells, whereas in other three cell lines they were all higher than 64 μM. Western blot were then analyzed in the presence of ZD6474, the phosphorylation of KIF5B-RET was reduced by ZD6474 in a concentration-dependent manner (Figure 6B). Consistent with these results, ZD6474 significantly inhibited the cell proliferation and colony formation of the KIF5B-RET-expressing A549 cells, and it exerted less effects on the control A549 cells (Figures 6C- 6E). These findings suggest that the suppression of the RET kinase activity reduces the proliferation of KIF5B-RET positive cells *in vitro*. Since STAT3 pathway plays an important role in mediating the KIF5B-RET promoted cell growth, we used some related inhibitors to investigate the effect on cell growth after down regulation of STAT3 signaling. U0126 is a MEK inhibitor which inhibits STAT3 Ser^727^ phosphorylation mediated through the Ras/Raf/MEK1/2/ERK1/2 pathway. PP1 is an inhibitor of Src-family tyrosine kinases which inhibits upstream kinases for the STAT3 Tyr^705^ phosphorylation. S3I-201 is an inhibitor of STAT3 transcription factor activation, dimerization, and gene transcription. Colony formation assays were performed in the presence or absence of the inhibitors U0126, PP1, or S3I-201. Significantly, all these inhibitors reduced the number and sizes of colonies of A549 cells carrying KIF5B-RET (Figure 7A). And the cell proliferation of A549 cells with KIF5B-RET also markedly decreased after the treatment of the STAT3 inhibitors S3I-201(Figure 7B).

## Discussion

In the era of personalized medicine, tailored treatment based on gene alterations, including gene mutations and gene fusions, called as “driver genes”, has become a standard practice [31]. Gene alterations encode signaling proteins which are crucial for cellular proliferation and survival, and drive tumor formation and sustain tumorigenesis. Partial chromosomal translocation and the corresponding gene fusion are not unusual among malignancies and may serve as a driving force for carcinogenesis. Thanks to refinements in cytogenetic techniques, an increasing number of gene fusions are being discovered in NSCLCs, such as ALK rearrangement and ROS1 rearrangement [5,6]. Most recently, the RET gene rearrangement has been identified and has been demonstrated to be a new driver mutation in a subset of lung adenocarcinomas in some studies [8,29]. In contrast to 13 fusion partner genes of RET in thyroid carcinoma, only KIF5B-RET, CCDC6-RET, TRIM33-RET and NCOA4-RET have been reported in lung cancer, and KIF5B-RET is the most commonly identified gene fusion in NSCLCs to date [29,32].

The overall prevalence of RET fusions is 1% to 2% in an unselected population of NSCLCs, however, this incidence increases substantially to 6% in never-smokers with lung adenocarcinomas that are pan-negative for other known driver mutations [13]. Based on the huge number of global lung cancer patients, 6% is not a small figure. The identification of RET fusion genes represent a new addition to the growing list of actionable drivers in lung cancers, add to the knowledge of the underlying factors behind this malignancy, and more importantly, it might change today’s therapeutic landscape in lung cancers.

It has been observed in our study that KIF5B-RET fusion protein has higher levels of phosphorylation and enhances cell proliferation significantly. On the other hand, in the report in Journal of Clinical Oncology, Wang et al. [29] found that all RET-positive adenocarcinomas had a small primary lesion but tended to present with N2 disease significantly more often than the other lung adenocarcinomas with small lesions (P < 0.024), which showed KIF5B-RET positive tumor may have stronger local invasion ability. The potential role of KIF5B-RET fusion genes in lung carcinogenesis deserves further study.

The KIF5B-RET fusion protein comprises the motor domain and the coiled-coil domain of KIF5B, and the juxtamembrane intracellular region of RET, including the entire tyrosine kinase domain. Because KIF5B is expressed ubiquitously, its active promoter may drive the expression of KIF5B-RET. Together with dimerization through the coiled-coil domain, the RET tyrosine kinase activity of the fusion protein may be activated aberrantly, thus facilitating oncogenesis in the lung. This hypothesis corroborates the oncogenic mechanism proposed for the fusion gene KIF5B-ALK, in which the coiled-coil domain of KIF5B is always preserved, and its constitutive expression in lung is believed to activate ALK and downstream oncogenic effects [30,33].

Although information on signal transduction downstream of the KIF5B-RET is still limited, it has been demonstrated that several tyrosine residues are phosphorylated in RET with GDNFs stimulation, which serve as docking sites recruits the Grb2-Gab1 and Grb2-Sos complexes that then activate the PI3K/AKT, RAS/MAPK, p38MAPK and c-Jun N-terminal kinase (JNK) pathways respectively, which promote cell proliferation and survival in some endocrine tumors [34]. In our study, KIF5B-RET fusion kinase was shown to possess marked oncogenic activity both *in vitro* and *in vivo*, and STAT3 signaling pathway might be the principal downstream mediator of the oncogenesis. Strong phosphorylation of STAT3 was presented in KIF5B-RET positive lung cancer cells. Here we provide several lines of evidence that show KIF5B-RET mediates continuous activation of STAT3. The fusion kinase could bind to STAT3, and directly phosphorylate and activate STAT3 Tyr^705^. It also can mediate activation of STAT3 Tyr^705^ in the JAKs/STAT3 dependent ways, and trigger Ser^727^ phosphorylation through the Ras/Raf/MEK_1/2_/ERK_1/2_ pathway. All in all, KIF5B-RET fusion protein regulates STAT3 activation at different levels which may target cyclinD1 and play a key role in oncogenesis.

Accumulating data shows that most tumors will depend on more than one signaling pathway for their growth and survival, which necessitates either the development of multitargeted agents or the combination of single targeted drugs to inhibit multiple signaling pathways or multiple steps in the same pathway [35]. In our study, different inhibitors were used to suppress multiple steps of the KIF5B-RET-STAT3 pathway, such as MEK inhibitor (U0126), JAKs or Src-family tyrosine kinases inhibitor (AG490 and PP1), STAT3 inhibitor (S3I-201) and multi-targeted agent (ZD6474). Significantly, all these inhibitors reduced the cell proliferation of KIF5B-RET positive lung cancer cells *in vitro*. However, the use of a combination of different agents will also be less convenient to the patient and can result in more dosing mistakes, so further basic and clinical studies are warranted to assess the optimize target inhibition.

## Conclusions

Our results have consolidated the role of KIF5B-RET fusion gene in the pathogenesis of NSCLC and identified STAT3 as a key mediator of the transforming activity of KIF5B-RET positive lung cancer cells. KIF5B-RET fusion protein regulates STAT3 activation at multilevels which may target cyclinD1 and play a key role in oncogenesis. Our results thus provide possible strategies for the treatment of KIF5B-RET positive lung cancer patients.

## Materials and methods

### Cell lines

A549, H1299, Beas-2b, and 293 T cell lines were all from the cell bank of Chinese academy of sciences. A549 and H1299 cells were cultured at 37°C in RPMI-1640 supplemented with 10% heat-inactivated FCS. Beas-2b and 293 T cells were cultured in DMEM with 10% FCS.

### Chemicals and antibodies

Different inhibitors of specific signal transduction pathways, including Vandetanib (ZD6474), U0126, PP1, AG490 and S3I-201, were purchased from Selleck. Phosphor-Ret(Tyr^905^), Ret, phospho-STAT3 (Tyr^705^), Phospho-STAT3(Ser^727^), STAT3, phospho-ERK1/2(Thr^202^/Tyr^204^), ERK_1/2_, glyceraledehyde-3-phosphatedehydrogenase (GAPDH), and anti-Flag antibodies were purchased from Cell Signaling Technology. STAT3 recombinant protein was purchased from Abnova.

### Sample collection

Primary lung cancers tissues were from Chinese patients who did not receive neoadjuvant therapy and who underwent resection at Zhejiang Provincial Cancer Hospital, Hangzhou, between 2008 and 2010. The corresponding non-neoplastic lung tissues were immediately frozen and stored at −80°C until assayed. Informed consent and ethics approval was obtained for research purposes. Ethics committee of the hospital approved the study.

### RT- PCR

Total RNA was extracted from lung cancer tissues or cultured cells with TRIzol Reagent (Invotrogen). Revert Aid First Strand cDNA Synthesis Kit (Fermentas) was used to construct the template cDNA for realtime PCR. The primer sequences for screening the KIF5B/RET fusion gene were as follows: forward primer, 5′-AGGAAATGACCAACCACCAG-3′, and reverse primer, 5′-TCCAAATTCGCCTTCTCCTA-3′. PCR was performed with initial denaturation at 95°C for5 min, followed by 40 cycles of amplification (at 98°C for 10 sec, 60°C for 15 sec, and 72°C for 3 min), and final extension at 72°C for 5 minutes.

### Western blot

Total protein lysates were obtained from cultured cells or tumor tissues using radio-immunoprecipitation assay. Cell extracts were subjected to sodium dodecyl sulfate-polyacrylamide gel electrophoresis (8% polyacrylamide gels) and then transferred to nitrocellulose membranes. The membranes were blocked overnight with TBS containing 0.1% Tween 20 (TBST) and 5% nonfat milk, and probed with the primary antibodies overnight at 4°C. After washing with TBST, the membranes were incubated with horseradish peroxidase (HRP)–conjugated secondary antibody for 1 h at room temperature, washed with TBST 3 times, and reacted with Super Signal West Pico chemiluminescent substrate (Pierce).

### Hematoxylin-eosin and immunohistochemical stain

Tumor samples were fixed in 10% neutral-buffered formalin for 24 h and embedded in paraffin. For hematoxylin-eosin stain, sections were reacted with hemalum for nuclear staining, which is followed by counterstaining with eosin for staining of other eosinophilic structures. For immunohistochemical stain, sections were reacted with primary antibodies for 24 h at 4°C, reacted with HRP–conjugated secondary antibody for 1 h, and diaminobenzidine (DAB) was used as the substrate to produce an observable brown color.

### Plasmids and lentiviral packaging

A full-length KIF5B-RET cDNA was cloned into lentiviral vector for constitutive gene expression (pLenti6.3-MCS-IRES2 vector, Invitrogen). Lentiviral vector was cotransfected with pLP1, pLP2, and pLP/VSVG packaging vectors (Invitrogen) into 293 T cells. A549 and Beas-2b cells were infected with empty, KIF5B-RET-FLAG -expressing lentiviruses, and were treated with Blastcidin (10 μg/ml) for 2 weeks.

### Colony formation assay

300 cells were cultured in a 6-well culture plate for 14 days. At the end of incubation, cells were fixed with methanol for 10 min and stained with crystal violet for 10 ~ 15 min. Three wells of cell colonies were scored (≥40 cells).

### Xenograft tumor model

Four-week-old athymic nude mice (BALB/c nude) were used in these studies with the approval of the institution’s ethics committee. 1 × 10^6^ control and A549 cells, carrying KIF5B-RET, were separately subcutaneously injected in the left and right flanks of mice. The tumors were measured 1 month later. The weight of the xenograft tumors was recorded and analyzed.

### Co-Immunoprecipitation (Co-IP)

Flag and STAT3 antibodies were used for Co-IP. Cell lysates were incubated with primary antibodies or control IgG overnight at 4°C and the immune complex was precipitated by the ProteinG Magnetic Beads (Millipore).The beads were then washed, boiled, and subjected to SDS-PAGE.

### Statistical analysis

Values are shown as means ± SEM. Statistical analysis was performed by Student’s t-test, and P value less than 0.05 was considered statistically significant.

## Abbreviations

NSCLC: Non-small cell lung cancer; LAD: Lung adenocarcinoma; KIF5B: Kinesin family member 5B gene; RET: Rearranged during transfection gene; GDNF: Glial derived neurotrophic factor; STAT3: Signal transducer and activator of transcription-3; JAK: Janus-like kinase; EGFR: Epidermal growth factor receptor; KRAS: Kirsten rat sarcoma; EML4-ALK: Echinoderm microtubule associated protein like 4 –anaplastic lymphoma receptor tyrosine kinase; TKI: Tyrosine kinase inhibitors.

## Competing interests

The authors declare that they have no competing interests.

## Authors’ contributions

YYQ was involved in designing and execution as well as supervision of work. SJC was involved in data analysis and the preparation of manuscript. ZYL, YFW, FTH and YZ were involved in different cell and animal based experiments. JXS, WH, ZDH and MZ were involved in lung cancers tissues collection and RT-PCR analysis. KW have done overall supervision of work. All authors read and approved the final version of manuscript.

## Supplementary Material

Additional file 1 Lung cancer cases screened for KIF5B-RET fusions. Click here for file
