# Correction to: KIF5B-RET fusion kinase promotes cell growth by multilevel activation of STAT3 in lung cancer

**DOI:** 10.1186/s12943-019-1093-0

**Published:** 2019-11-19

**Authors:** Yingying Qian, Shoujie Chai, Zuyu Liang, Yongfang Wang, You Zhou, Xia Xu, Chenchen Zhang, Min Zhang, Jingxing Si, Feiteng Huang, Zhangdan Huang, Wei Hong, Kai Wang

**Affiliations:** 1 Department of Respiratory Medicine, Second Affiliated Hospital, School of Medicine, Zhejiang University, Hangzhou, 310009 China; 2 Department of Medical Oncology, Zhejiang Cancer Hospital, Hangzhou, 310022 China

**Correction to: Molecular Cancer (2014) 13:176.**

**http://www.molecular-cancer.com/content/13/1/176**

After the publication of this work [1], the authors noticed errors in Fig. 4. The GAPDH data in panels A and C were mistakenly cut out. The incorrect band of t-ERK was included in Fig. 4B. The p-RET and t-RET panels C and D were mistakenly switched. The corrected panels for Fig. 4 are below. We apologize for any inconvenience caused, but the errors do not change the conclusion or discussion of our article. 
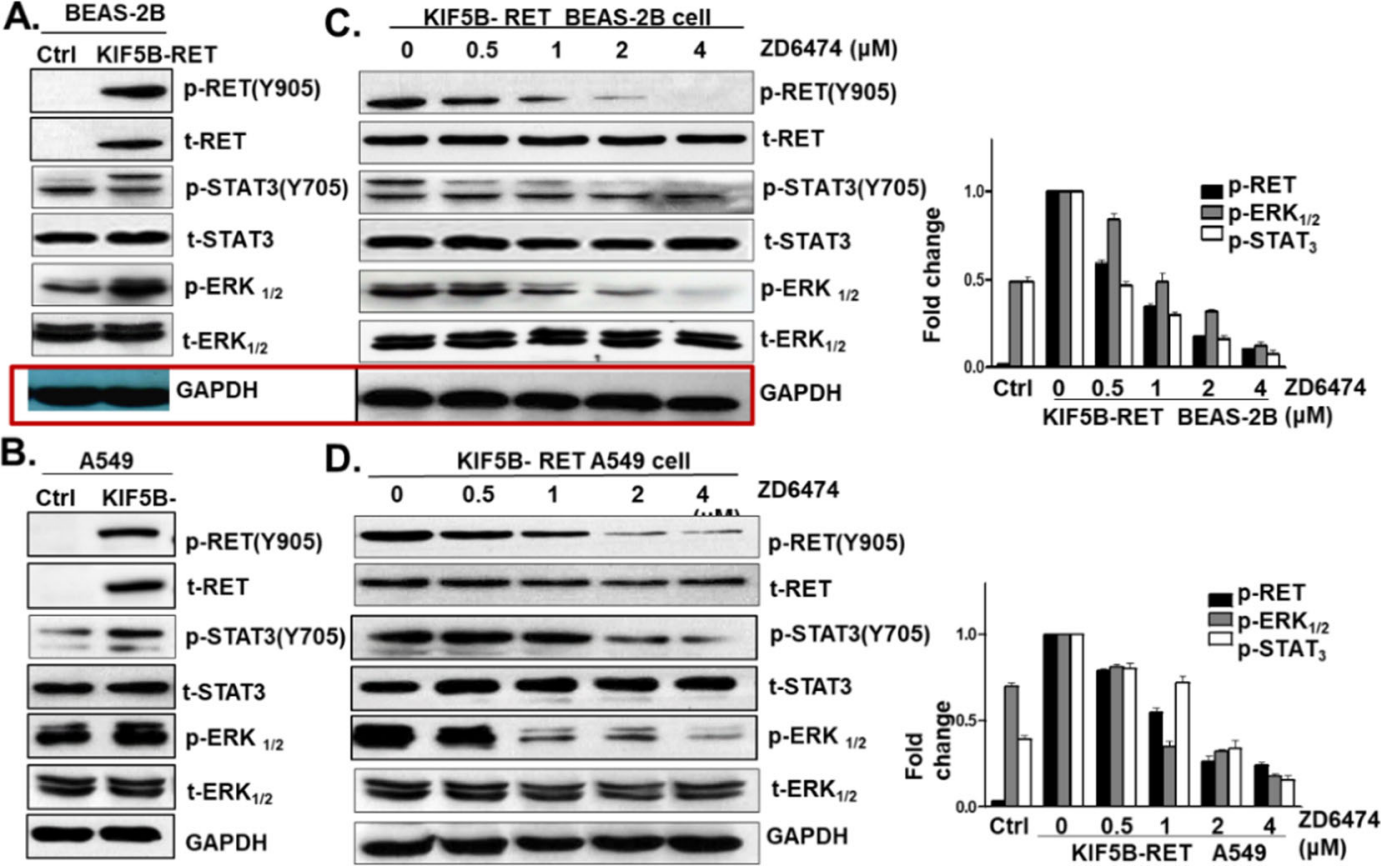
 
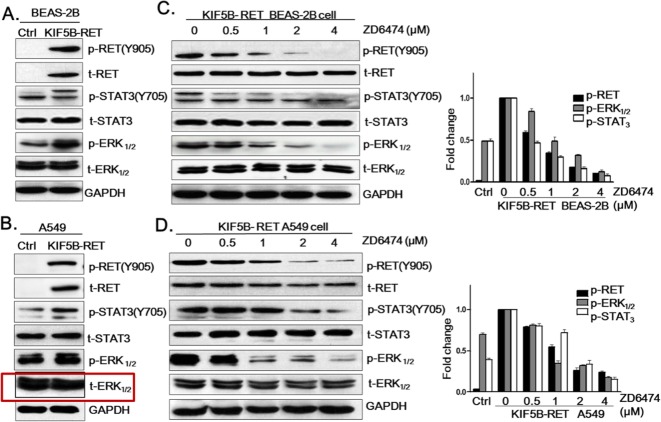
 
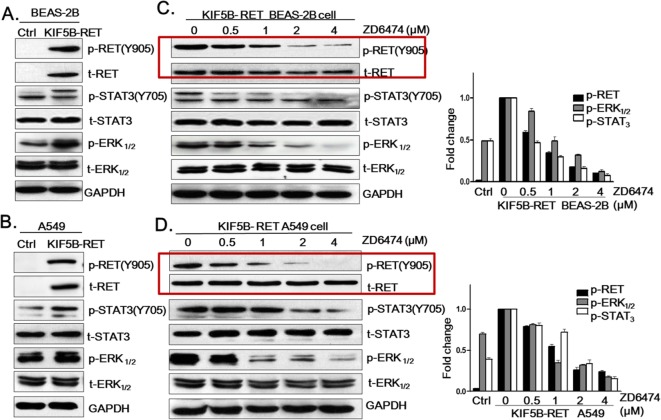


## References

[1] YingYing Qian, Shoujie Chai, Zuyu Liang, Yongfang Wang, You Zhou, Xia Xu, Chenchen Zhang, Min Zhang, Jingxing Si, Feiteng Huang, Zhangdan Huang, Wei Hong and Kai Wang. (2014). KIF5B-RET fusion kinase promotes cell growth by multilevel activation of STAT3 in lung cancer. Molecular Cancer 2014, 13:176. http://www.molecular-cancer.com/content/13/1/176. 10.1186/1476-4598-13-176 PMC4114102 25047660

