# Supplementary material for: KIF5B-RET fusion kinase promotes cell growth by multilevel activation of STAT3 in lung cancer
Source: Mol Cancer. 2014 Jul 21;13:176. doi: 10.1186/1476-4598-13-176 (PMC4114102; doi:10.1186/1476-4598-13-176)
Supplement: Additional file 1 — Lung cancer cases screened for KIF5B-RET fusions. [file 1476-4598-13-176-S1.pptx]

## Slide 1
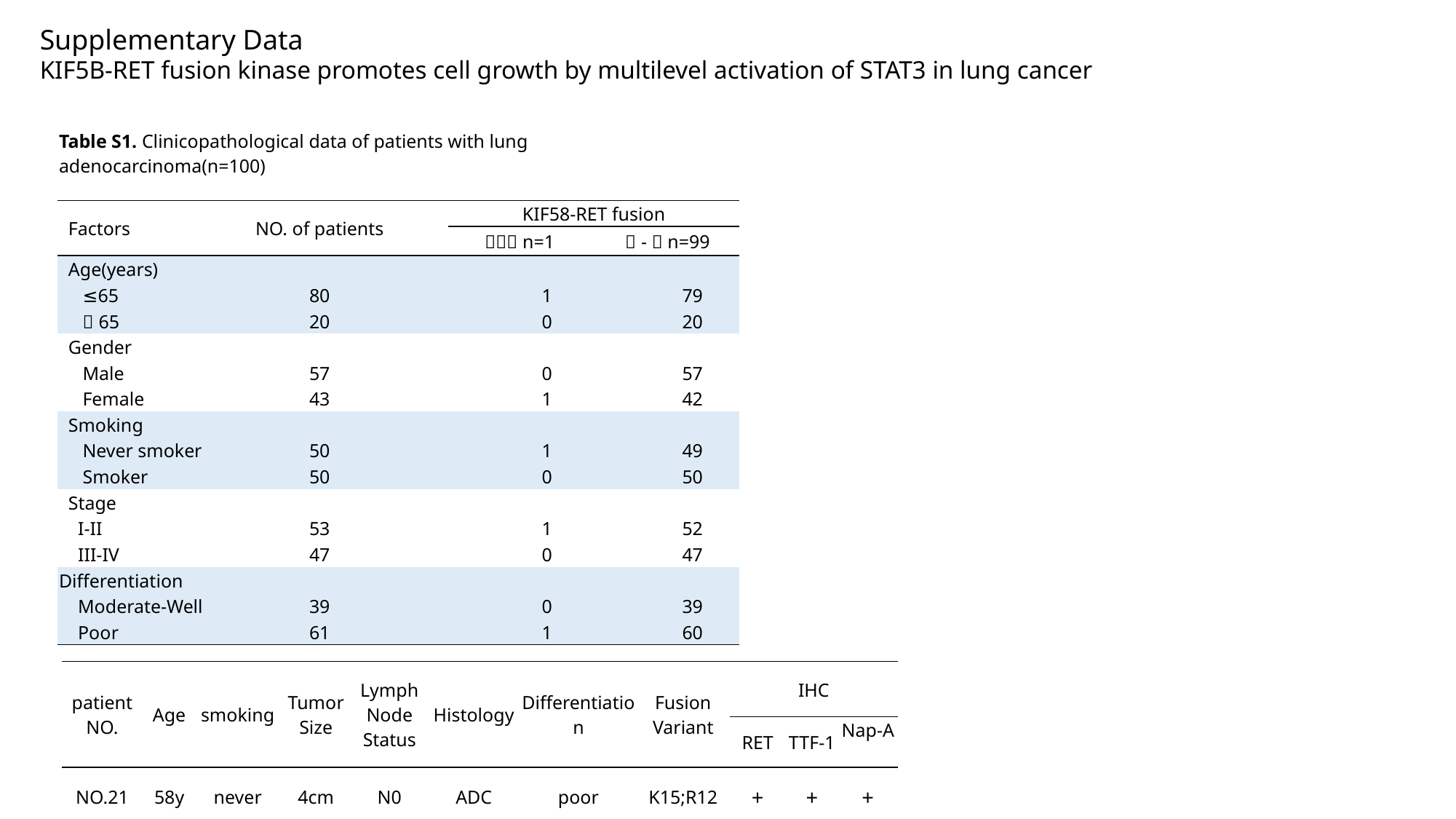

Supplementary Data
KIF5B-RET fusion kinase promotes cell growth by multilevel activation of STAT3 in lung cancer
| Table S1. Clinicopathological data of patients with lung adenocarcinoma(n=100) | | | | | | | | | |
| --- | --- | --- | --- | --- | --- | --- | --- | --- | --- |
| Factors | | NO. of patients | | | KIF58-RET fusion | | | | |
| | | | | | （＋）n=1 | | （-）n=99 | | |
| Age(years) | | | | | | | | | |
| ≤65 | | 80 | | | 1 | | | 79 | |
| ＞65 | | 20 | | | 0 | | | 20 | |
| Gender | | | | | | | | | |
| Male | | 57 | | | 0 | | | 57 | |
| Female | | 43 | | | 1 | | | 42 | |
| Smoking | | | | | | | | | |
| Never smoker | | 50 | | | 1 | | | 49 | |
| Smoker | | 50 | | | 0 | | | 50 | |
| Stage | | | | | | | | | |
| I-II | | 53 | | | 1 | | | 52 | |
| III-IV | | 47 | | | 0 | | | 47 | |
| Differentiation | | | | | | | | | |
| Moderate-Well | | 39 | | | 0 | | | 39 | |
| Poor | | 61 | | | 1 | | | 60 | |
| | | | | | | | | | |
| Table S2. Clinicopathologic details of KIF5B-RET positive lung cancer patient | | | | | | | | | | |
| --- | --- | --- | --- | --- | --- | --- | --- | --- | --- | --- |
| patient NO. | Age | smoking | Tumor Size | Lymph Node Status | Histology | Differentiation | Fusion Variant | IHC | | |
| | | | | | | | | RET | TTF-1 | Nap-A |
| NO.21 | 58y | never | 4cm | N0 | ADC | poor | K15;R12 | + | + | + |

## Slide 2
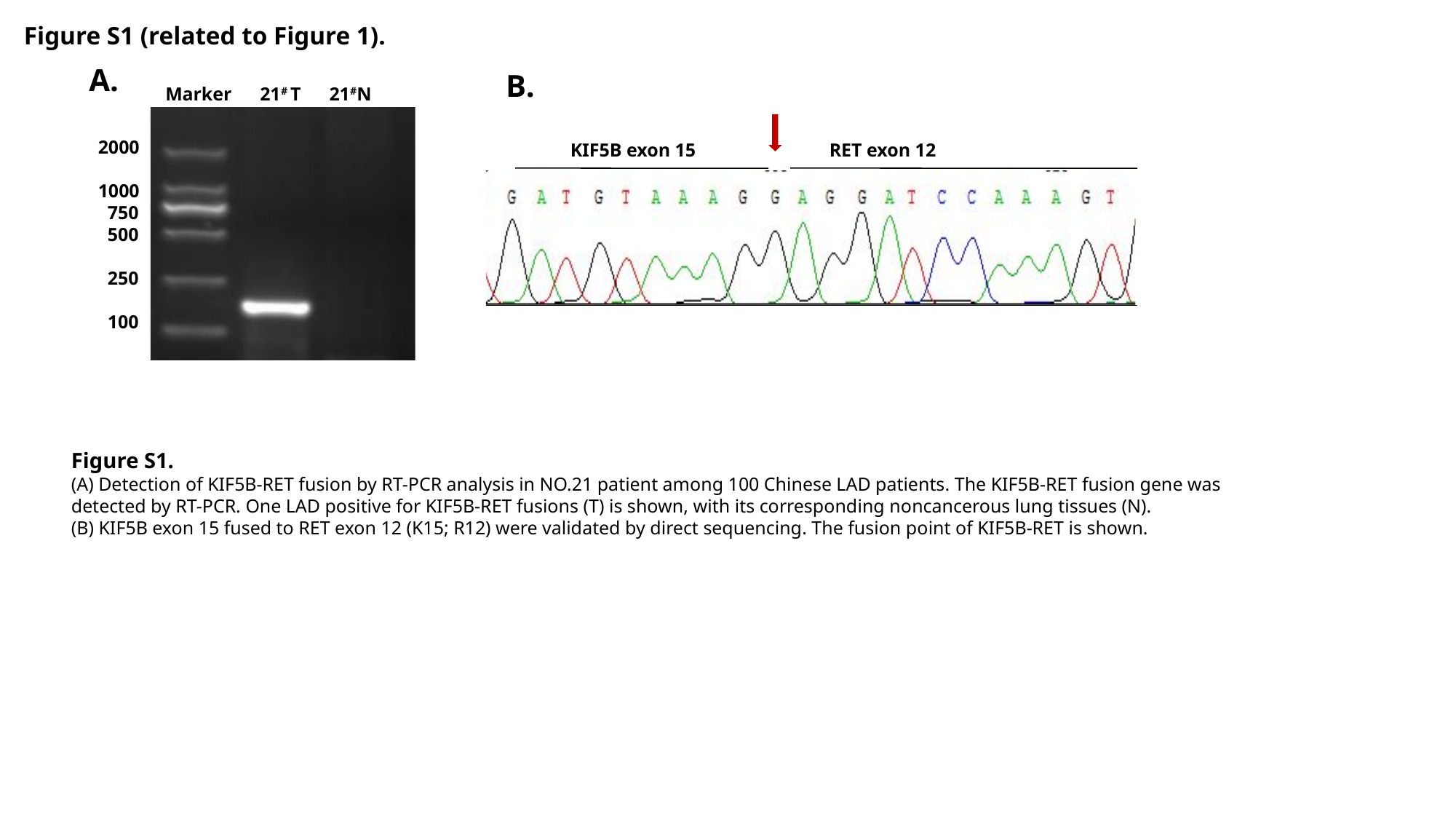

Figure S1 (related to Figure 1).
A.
 Marker 21# T 21#N
2000
1000
 750
 500
 250
 100
B.
KIF5B exon 15
RET exon 12
Figure S1.
(A) Detection of KIF5B-RET fusion by RT-PCR analysis in NO.21 patient among 100 Chinese LAD patients. The KIF5B-RET fusion gene was detected by RT-PCR. One LAD positive for KIF5B-RET fusions (T) is shown, with its corresponding noncancerous lung tissues (N).
(B) KIF5B exon 15 fused to RET exon 12 (K15; R12) were validated by direct sequencing. The fusion point of KIF5B-RET is shown.
